# Magnetic Resonance Cholangiopancreatography for Cholangiopancreatic Duct Imaging in Dogs

**DOI:** 10.1111/vru.70008

**Published:** 2025-01-18

**Authors:** Reina Fujiwara, Kie Yamamoto, Masahiro Yamasaki, Koichi Ohno

**Affiliations:** ^1^ Division of Companion Animal Internal Medicine, Veterinary Teaching Hospital, Faculty of Agriculture Iwate University Morioka Japan; ^2^ Veterinary Medical Center Graduate School of Agricultural and Life Sciences The University of Tokyo Tokyo Japan; ^3^ Laboratory of Veterinary Small Animal Internal Medicine Department of Veterinary Medicine Faculty of Agriculture Iwate University Morioka Japan; ^4^ Animal Medical Center, Peco Tokyo Japan

**Keywords:** biliary duct, dog, MRCP, MRI, pancreatic duct

## Abstract

Ultrasonography is often used to diagnose biliary diseases in dogs; however, it is difficult to delineate the entire bile and pancreatic ducts. Various imaging techniques for bile and pancreatic ducts have been attempted to overcome this problem. Magnetic resonance cholangiopancreatography (MRCP) is often used to evaluate the bile and pancreatic ducts in humans with obstructive jaundice, but very few reports exist on its usage in dogs. This study was designed as a prospective observational study to assess the feasibility and effectiveness of MRCP for visualizing the bile and pancreatic ducts in nondiseased dogs. Therefore, this study aimed to evaluate the visibility of the bile and pancreatic ducts through MRCP imaging using a 3.0 T‐MRI system in dogs with no signs of hepatic, biliary, and pancreatic diseases. The detection rate for each anatomical structure was evaluated, with the highest observed in the gallbladder (100%), followed by the common bile duct (80%), cystic duct (70%), pancreatic ducts in the left and right lobe of the pancreas (70%), left and right hepatic ducts (60%), accessory pancreatic ducts (60%), and major pancreatic duct (40%). MRCP is a promising noninvasive imaging technique that can promptly and accurately visualize bile and pancreatic ducts in dogs without being influenced by the skill of the operator.

## 1 Introduction

Biliary diseases in dogs and cats include cholecystitis, cholangitis, cholelithiasis, gallbladder mucoceles, and gallbladder aplasia [1, 2, 3, 4, 5, 6, 7, 8, 9, 10]. In these diseases, imaging of the biliary system and pancreatic ducts is important for diagnosis, severity assessment, and treatment decisions [6, 9, 11]. Ultrasonography is a suitable screening test because it does not involve radiation exposure, is not invasive, and the equipment is widely available. However, it is difficult to evaluate the entire biliary system and pancreatic duct using ultrasonography, and it depends on the skill of the operator [9, 12, 13]. In dogs and cats, in addition to ultrasonography, various imaging modalities, including cholecystography, cholangiography, CT, and endoscopic retrograde cholangiopancreatography (ERCP) have been attempted to overcome this problem [14, 15, 16, 17, 18, 19].

Magnetic resonance cholangiopancreatography (MRCP) visualizes the bile and pancreatic ducts by using heavily T2‐weighted images to produce stationary or extremely slow‐flowing water components with a high signal, whereas other structures have almost no signal [20, 21]. In humans, MRCP has become the new examination technique that replaces the diagnostic ERCP—which had previously been the gold standard—because MRCP does not use contrast agents, is noninvasive, and has made significant advances in both software and hardware to obtain high‐quality images [13, 22–24]. MRCP can visualize both the upstream and downstream of the obstruction and provides a great deal of information, such as the presence of exudates associated with inflammation, that cannot be obtained using ERCP [13, 25, 26]. Currently, it is becoming popular as an imaging test in patients with obstructive jaundice who do not require early endoscopic intervention [26].

The first report of MRCP in cats was published by Marolf et al. in 2011 [27]. Five healthy cats underwent MRI using various sequences of hepatobiliary pancreatic imaging in combination with secretin stimulation, including MRCP [27]. The same authors performed similar imaging in 10 cats with cholangitis and pancreatitis in 2013 and comprehensively evaluated the pancreas and pancreatic ducts using various sequences, including MRCP [12]. However, while dogs have been evaluated using cadavers, there have been very few reports of MRCP [28].

The purpose of this study was to perform MRCP imaging in dogs with no signs of hepatic, biliary, and pancreatic diseases using a 3.0T‐MRI system to evaluate the visibility of the biliary system and pancreatic ducts and to measure the lumen diameter. We hypothesized that both the biliary and pancreatic ducts could be visualized in dogs but that the pancreatic ducts, being thinner, would be less visible than the biliary system.

## 2 Materials and Methods

All dogs were client‐owned and underwent MRCP between January 2019 and March 2020 at the Veterinary Medical Center of the University of Tokyo. This was designed as a prospective observational study to evaluate the visibility of both the bile duct and pancreatic duct in dogs using MRCP imaging. The primary objective was to visualize the bile duct for diagnosis and treatment planning of obstructive cholestasis; however, we also evaluated the pancreatic ductal system to observe changes in the pancreatic duct during bile duct obstruction. The patients were scheduled to undergo an MRI scan of the central nervous system for the diagnosis of neurological diseases or for medical examination. Of these patients, those with no abnormal blood tests or ultrasound examinations and no relevant medical history of the liver, biliary system, or pancreas were included. MRCP imaging was performed after imaging of the central nervous system. This study did not require approval from the Institutional Animal Care and Use Committee because it involved clinical cases with client‐owned animals, and no experimental procedures were performed. All procedures were conducted in accordance with the ethical guidelines for the use of animals in clinical research, and informed consent was obtained from the owners of all dogs involved in the study.

Anesthesia was induced with propofol 2–6 mg/kg IV (propofol intravenous injection 1% “Maruishi”; Maruishi Pharmaceutical, Osaka, Japan). An endotracheal tube was placed, and anesthesia was maintained with isoflurane 1.5–2.0% (ds Isoflurane; Bussan Animal Health, Osaka, Japan) and oxygen under mechanical ventilation. The ventilator was adjusted to prevent spontaneous respiration at a rate of 10–15 breaths during the MRI scan.

A 3.0T‐MR system (Vantage Galan 3T; Canon Medical Systems, Tochigi, Japan) and a 16‐channel flexible coil were used. The dogs were held in ventral recumbency, and the ventilator was set to a respiratory rate of 10–15/min. Prior to MRCP, T2 W was performed on the transverse and dorsal planes of the cranial abdomen. Based on the obtained images, the field of view included the hepatic hilum, the entire gallbladder, and the pancreas. The acquired sections were either coronal or slightly oblique to the coronal plane. Heavily T2 W imaging was obtained using respiratory‐triggering multi‐slice 3D HASTE. The parameters for MRCP sequence at our institute are as follows: effective TE = 611 msec; flip angle = 90°; matrix = 288 × 404; FOV = 180 × 150–250 mm (depending on the area of interest); slice thickness = 1.0 mm; in‐plane image resolution = 0.70 × 0.62 mm; echo train length = 150; echo spacing = 6.5 ms; readout bandwidth = 488 Hz/Px; and acquisition time = 3–5 min (depends on respiratory rate).

All measurements were performed by an experienced investigator (R.F.) using the DICOM imaging software (OsiriX MD, PIXMEO; Bernex, Switzerland). Visibility of the gallbladder, cystic duct, common bile duct, left hepatic ducts, right hepatic duct, major pancreatic duct, accessory pancreatic duct, pancreatic duct within the left lobe of the pancreas, and pancreatic duct within the right lobe of the pancreas were evaluated. Multiplanar reconstruction and maximum intensity projection (MIP) were performed as required to evaluate the anatomical structure and continuity of the biliary system and pancreatic ducts. The visibility of each lumen was evaluated using four grades: Excellent, Good, Fair, and Poor (excellent = the entire structure was visible, and the contrast was good; good = the entire structure was visible but the contrast was poor, or more than half of the structure was visualized and the contrast was good; fair = less than half of the structure was visualized and the contrast was poor; poor = the structure could not be seen). The sum of the Excellent, Good, and Fair results was used as the percentage of the detection rate. Furthermore, the maximum diameters of the cystic duct, common bile duct, left hepatic duct, right hepatic duct, major pancreatic duct, accessory pancreatic duct, pancreatic duct within the left lobe of the pancreas, and pancreatic duct within the right lobe of the pancreas were also measured. The average of two measurements taken at intervals of at least 1 week by the same reader for the visual evaluation was calculated.

The median and standard deviation of the maximum diameter for each site were calculated.

EZR was used to calculate the standard deviation (EZR; Saitama Medical Center, Jichi Medical University, Saitama, Japan) [29].

## 3 Results

**FIGURE 1:**
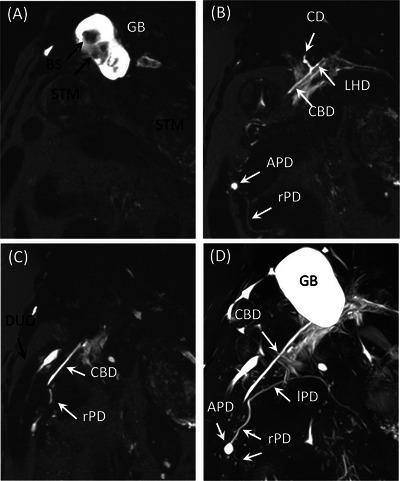
MRCP images of Dog 7. All structures except the major pancreatic duct are excellent. A–C, Partial MIP images with a thickness of 3 mm. D, MIP image. APD, accessory pancreatic duct; BS, bile sludge; CBD, common bile duct; CD, cystic duct; DUO, duodenum; GB, gallbladder; LHD, left hepatic duct; lPD, pancreatic duct in the left lobe; MIP, maximum intensity projection; rPD, pancreatic duct in the right lobe; STM, stomach.

Ten dogs were included in this study (Supporting Information S1). The breeds included three each of Chihuahuas and Toy poodles, two Miniature dachshunds, and one each of Labrador retriever and mixed‐breed dog. There were three intact and four castrated male dogs, and one intact and two spayed female dogs with a median weight of 3.7 (2.1–23.8) kg and a median age of 10.0 (7.0–14.8 years). MRI examinations were performed for one epileptic seizure, eight spinal cord symptoms, and one follow‐up for brain hemorrhage. The MRI diagnoses for these cases were intervertebral disc herniation (6 dogs), brain tumor (1 dog), atlantoaxial instability (1 dog), degenerative lumbosacral stenosis (1 dog), and microcerebral hemorrhage (1 dog). The scan time associated with MRCP, including the acquisition of positioning image, was less than 10 min. The MRI was performed safely without any complications. Respiratory‐gated imaging could be performed satisfactorily on all dogs, and no artifacts secondary to body movement or breathing were observed in the obtained images. In all patients, the contrast between the bile/pancreatic juice and surrounding tissue was good, and the anatomical structures were well delineated (Figure 1A–D).

**FIGURE 2:**
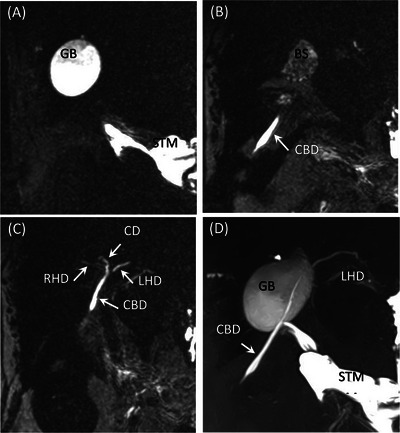
MRCP images of Dog 2. GB, CD, LHD, RHD, and CBD are Excellent, whereas MPD, APD, lPD, and rPD are all Poor. A–C, Partial MIP images with a thickness of 5 mm. D, MIP image. APD, accessory pancreatic duct; BS, bile sludge; CBD, common bile duct; CD, cystic duct; DUO, duodenum; GB, gallbladder; MPD, major pancreatic duct; LHD, left hepatic duct; lPD, pancreatic duct in the left lobe; MIP, maximum intensity projection; RHD, right hepatic duct; rPD, pancreatic duct in the right lobe; STM, stomach.

The highest detection rate and visibility were observed in the gallbladder in all dogs, with a detection rate of 100%. Of these, 80% (8/10) were Excellent and 20% (2/10) were Good. After the gallbladder, the common bile duct was the next well‐detected structure with 80% (excellent 60%, good 10%, fair 10%), followed by the cystic duct with 70% (excellent 40%, good 20%, fair 10%). The pancreatic ducts in the left and right lobes of the pancreas also had the same 70% detection rate as the cystic duct, but Excellent and Good were less common, whereas Fair was more common. The left and right hepatic ducts and accessory pancreatic ducts had a 60% detection rate, whereas the major pancreatic ducts had the lowest detection rate of 40% (Table 1, Figure 2A–D). The diameters of each lumen are listed in Table 1. Overall, larger lumens tended to have better delineation rates. However, the pancreatic duct had the lowest detection rate and visibility despite its median size of 1.0 mm.

**TABLE 1:** Detection rate for each part, visibility, and maximum diameter of the lumen.

	Detection rate (%)	Visibility (number of dogs)				Maximum diameter of lumen (mm)	
		Excellent	Good	Fair	Poor	Median	Range
GB	100	8	2	0	0	―	―
CD	70	4	2	1	3	1.6	1.5–2.5
LHD	60	2	2	2	4	0.8	0.7–1.5
RHD	60	2	2	2	4	0.9	0.4–1.2
CBD	80	6	1	1	2	1.4	0.9–3.0
MPD	40	0	0	4	6	1.0	0.8–1.2
APD	60	2	2	2	4	1.5	0.8–4.5
PD in the left lobe	70	2	1	4	3	0.7	0.5–1.4
PD in the right lobe	70	2	0	5	3	0.7	0.5–1.5

Abbreviations: GB, gall bladder; CD, cystic duct; LHD, left hepatic duct; RHD, right hepatic duct; CBD, common bile duct; MPD, major pancreatic duct; APD, accessory pancreatic duct.

## 4 Discussion

MRCP is an imaging technique used to delineate the biliary tree and pancreatic ducts with high contrast by revealing extremely slow‐flowing or stagnant fluids such as bile and pancreatic juice, resulting in high signal intensity, and the liver, pancreas, and surrounding structures resulting in low signal intensity [20, 21, 30]. In this study, we used the 3.0T‐MRI system to obtain MRCP in dogs and were able to comprehensively delineate the biliary system and pancreatic ducts. MRCP can be performed in coronal or oblique coronal sections in a single‐slice several centimeters thick (2D) or in continuous multi‐slice sections (3D), each of which has its own advantages and disadvantages [21, 31]. In the present study, we performed respiratory‐synchronized imaging under general anesthesia. Furthermore, considering various future 3D image processing methods such as MIP and volume rendering, a 3D method for obtaining continuous multi‐slice cross‐sections was selected.

In this study, the entire biliary system and pancreatic ducts could not be fully visualized in all dogs; however, there were some dogs in which the entire system and ducts were successfully visualized. This is the same in healthy human volunteers. In humans, the detection rate by MRCP is as high as 90–100% for the gallbladder, 75–90% for the cystic duct, and 90% for the intrahepatic bile duct [20, 32]. In addition, the normal pancreatic duct system detection rates were 100%, 93%, and 75–90% for the major pancreatic duct, Santorini duct, and cranial branch pancreatic duct, respectively [20, 33]. In the present study, the percentages of each site mentioned were 100% for the gallbladder, 80% for the common bile duct, 70% for the cystic duct and left and right pancreatic ducts, 60% for the left and right hepatic ducts and accessory pancreatic ducts, and 40% for the major pancreatic duct. The biliary system was relatively well‐delineated, whereas the major pancreatic duct was poorly delineated, which was attributed to its narrow diameter. However, the pancreatic ducts had a lower detection rate despite being larger in diameter than the right and left hepatic ducts and pancreatic ducts in the left and right lobes of the pancreas. This was more likely because, unlike humans and cats, in whom the main excretion route for pancreatic juice is the major pancreatic duct, in dogs, the main excretion route is the accessory pancreatic duct, so the pancreatic duct was more difficult to visualize than the accessory pancreatic duct [11, 34].

In addition to MRCP, imaging of the biliary system and pancreatic ducts includes radiography, CT, ERCP, and nuclear scintigraphy, which are combined with angiography, oral contrast media, transvenous cholangiography, and percutaneous cholecystography [14–19, 35–38]. Compared with other modalities, MRCP has disadvantages such as the need for anesthesia (unlike in humans), breath‐hold and breath‐synchronized imaging, the need for a high‐field MR system, and the high cost of examination.

However, MRCP is not affected by gases in the digestive tract, which is a problem with ultrasound examinations and does not depend on the skill of the operator [39]. Furthermore, unlike CT cholangiography, which uses iodine‐based contrast agents administered percutaneously or intravenously, it is not affected by liver function or the ability to excrete bile [40, 41].

Therefore, MRCP does not require a waiting period after contrast administration. Even in the presence of hyperbilirubinemia or bile duct obstruction, imaging is possible before and after complete biliary obstruction. The disadvantage of MRCP is that it is a static evaluation, and unlike CT cholangiography, it does not allow for the dynamic evaluation of bile flow or identification of the site of leakage. In addition, when the amount of ascites is large, both the ascites and the biliary system show high signals, making it difficult to identify the biliary system [42].

In veterinary medicine, MRCP is not a suitable substitute for ultrasound examination in terms of convenience, anesthesia, or cost. However, it allows continuous visualization of the entire biliary system, comprehensive evaluation of the biliary system and pancreatic ducts, and diagnosis of nonliquid retention, which are difficult to achieve with ultrasound examination. In dogs, when the extrahepatic biliary system is dilated, the causes are diverse and include obstruction by gallstones, narrowing of the common bile duct or large duodenal papillae, and obstruction of the common bile duct by mucins in the gallbladder mucocele [1, 2, 3, 4, 5, 6]. When surgery is required for extrahepatic bile duct obstruction, identification of the cause and site of the obstruction is important in selecting the surgical procedure [43]. For example, MRCP can be used to detect mucin in the hepatic and common bile ducts within 10 min. Based on the MRCP results, we could determine the necessity of washing the common bile duct when cholecystectomy is performed for a patient with a gallbladder mucocele without rupture. Thus, MRCP may be useful not only for diagnosis but also for surgical planning.

## 5 Limitations

The limitation of this study is that image evaluations were conducted by a single observer, preventing the assessment of inter‐observer reliability, which is key for ensuring consistency and generalizability. This also increases the risk of subjective bias and limits reproducibility. Including multiple observers and measuring interobserver agreement would enhance reliability in future studies.

Other limitations of this study include the limited number of dogs and their predominantly small size. While larger dogs may improve visualization due to thicker bile and pancreatic ducts, they may also present challenges such as reduced signal from deeper organs and decreased spatial resolution due to larger coils and field of view. Future studies should consider including a wide range of dog sizes to address these limitations.

## 6 Conclusion

MRCP is a promising noninvasive diagnostic imaging technique that can promptly and accurately depict the biliary and pancreatic duct systems (except for the major pancreatic duct) in dogs, without being affected by the skill of the operator.

## Conflicts of Interest

The authors declare no conflicts of interest.

## Previous Presentation or Publication Disclosure

The authors declare that this study was not previously presented at a scientific meeting.

## Reporting Checklist Disclosure

This study was conducted in accordance with the STROBE (Strengthening the Reporting of Observational Studies in Epidemiology) guidelines for reporting observational studies.

## Supporting information



Supporting Information

## Data Availability

The data supporting the results of this study are available from the corresponding author upon reasonable request.
