# Supplementary material for: Magnetic Resonance Cholangiopancreatography for Cholangiopancreatic Duct Imaging in Dogs
Source: Vet Radiol Ultrasound. 2025 Jan 18;66(1):e70008. doi: 10.1111/vru.70008 (PMC11742707; doi:10.1111/vru.70008)
Supplement: Supplementary file 1 — Supporting Information [file VRU-66-0-s001.docx]

**Supplement 1**

**Signalment and MRI diagnosis of the subjects**

| No. | Breed | Age | Sex | Body weight (kg) | MRI  diagnosis |
| --- | --- | --- | --- | --- | --- |
| 1 | Chihuahua | 7y2m | CM | 2.6 | IVDH |
| 2 | Chihuahua | 9y0m | SF | 3.4 | IVDH |
| 3 | Chihuahua | 13y4m | CM | 3.8 | IVDH |
| 4 | Toy Poodle | 7y1m | F | 2.1 | AAI |
| 5 | Toy Poodle | 9y8m | M | 3.3 | IVDH |
| 6 | Toy Poodle | 11y0m | CM | 8.3 | IVDH |
| 7 | Miniature Dachshund | 14y3m | SF | 4.0 | Brain hemorrhage |
| 8 | Miniature Dachshund | 14y9m | CM | 4.2 | Brain  tumor |
| 9 | Mix | 10y1m | M | 3.5 | IVDH |
| 10 | Labrador retriever | 9y11m | M | 23.8 | DLSS |

M, intact male; CM, castrated male; F, intact female; SF, spayed female; IVDH, intervertebral disc herniation; AAI, atlantoaxial instability; DLSS, degenerative lumbosacral stenosis
